# Cholangioscopy-assisted partial stent-in-stent method useful for bilateral biliary drainage of hilar cholangiocarcinoma

**DOI:** 10.1055/a-2218-2972

**Published:** 2023-12-21

**Authors:** Hideaki Kazumori, Yasuhiko Ohno, Kousaku Kawashima

**Affiliations:** 1 13838 Department of Gastroenterology, Matsue Seikyo General Hospital, Matsue, Japan; 2 2nd Unit of Internal Medicine, Shimane University, School of Medicine, Izumo, Japan

Bilateral drainage with the use of a partial stent-in-stent method is well known to resolve bile duct obstruction associated with a hilar cholangiocarcinoma. Unfortunately, the results are sometimes unsatisfactory because of the difficulty passing the guidewire through the targeted bile ducts, with reported success rates ranging from 80–95% 1 2 3 . Presented here is a novel cholangioscopy method.

A 94-year-old man with hilar cholangiocarcinoma presented with jaundice, and endoscopic retrograde cholangiography (ERCP) showed a high degree of hilar bile duct stenosis ( Fig. 1 **a** ). The guidewire failed to pass through the right hepatic duct and biliary drainage resulted in insertion into only the left hepatic duct with a plastic stent ( Fig. 1 **b** ). Thus, for jaundice, use of a partial stent-in-stent method to insert a self-expandable metallic stent (SEMS) with cholangiography was planned ( Video 1 ). The right hepatic duct was not fully contrasted, and cholangioscopy (SpyScope DSII; Boston Scientific Corp., Marlborough, Massachusetts, USA) was performed to locate the entrance. However, stenosis of the common hepatic blocked cholangioscope advancement ( Fig. 2 **a** ). Following balloon dilatation, the cholangioscope was successfully advanced and the right hepatic duct entrance found ( Fig. 2 **b** ), with guidewire insertion performed ( Fig. 3 **a** ). A SEMS, with a diameter of 10 mm and length of 60 mm (ZEOSTENT V; Zeon Medical Inc., Tokyo, Japan) was then inserted and expanded, followed by cholangioscope insertion into the detained SEMS, with a mesh connected directly to the left hepatic duct chosen ( Fig. 2 **c** ). The guidewire was passed through the mesh ( Fig. 3 **b** ), then a second SEMS (ZEOSTENT V) with same diameter and length was inserted and expanded ( Fig. 3 **c** ).

**Fig. 1:**
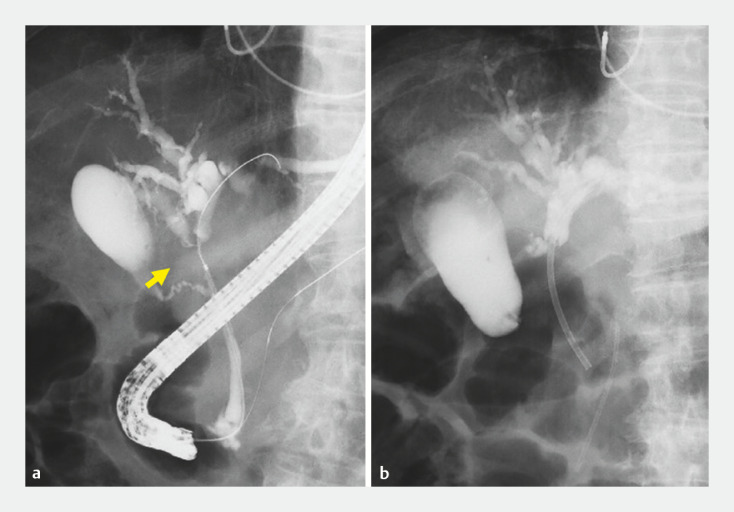
Endoscopic retrograde cholangiography (ERCP) images of hilar cholangiocarcinoma. **a** A high degree of hilar bile duct stenosis was observed. **b** Biliary drainage resulted in insertion only into the left hepatic duct.

Cholangioscopy-assisted bilateral biliary drainage method using partial stent-in-stent. The guidewire is inserted into the targeted bile duct revealed by cholangioscopy, followed by insertion of a self-expandable metal stent.Video 1

**Fig. 2:**
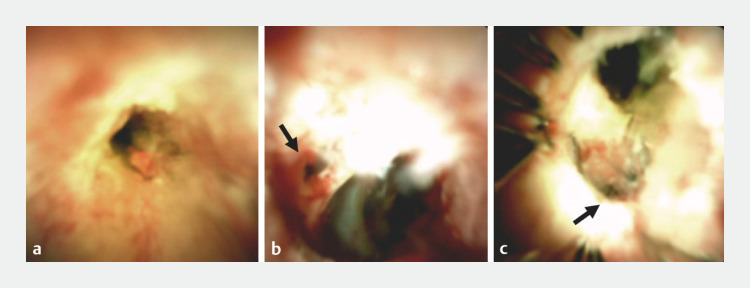
Cholangioscopy images. A SpyScope DS II was used. **a** Malignant stenosis of common bile duct. **b** Entrance to right bile duct. **c** Entrance to left bile duct. The self-expandable metallic stent (SEMS) mesh leading straight to the left bile duct was chosen.

**Fig. 3:**
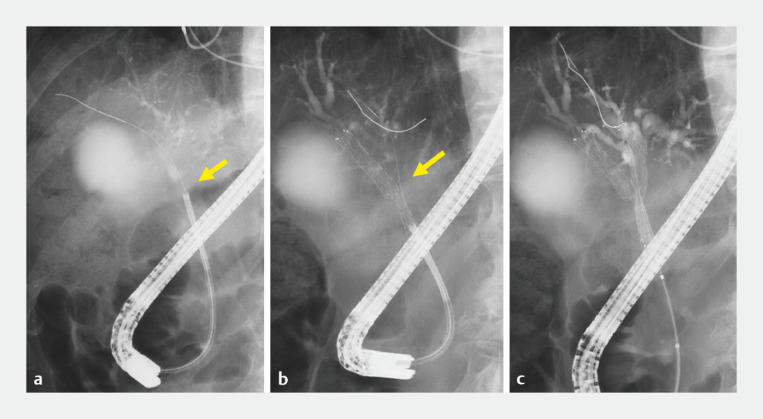
ERCP images of partial stent-in-stent. Two SEMSs were inserted, each with a diameter of 10 mm and length of 60 mm. **a** Insertion of guidewire into right hepatic duct shown by cholangioscopy. **b** Insertion of guidewire through SEMS mesh into the left hepatic duct shown by cholangioscopy. **c** Bilateral biliary drainage was successfully performed by use of a partial stent-in-stent method.

In hilar cholangiocarcinoma cases with a high degree of bile duct stenosis, guidewire insertion into the targeted bile duct is difficult. Notably, placement of the second SEMS was difficult in the present patient and required searching for the mesh leading to the targeted bile duct, passage of which was often obstructed by the mesh itself. In the present case, cholangioscopy revealed the entrance location. When biliary drainage is difficult, cholangioscopy findings can help resolve such problems.

Endoscopy_UCTN_Code_TTT_1AR_2AZ

## Notes

Endoscopy E-Videos https://eref.thieme.de/e-videos
*E-Videos* is an open access online section of the journal *Endoscopy* , reporting on interesting cases and new techniques in gastroenterological endoscopy. All papers include a high-quality video and are published with a Creative Commons CC-BY license. Endoscopy E-Videos qualify for HINARI discounts and waivers and eligibility is automatically checked during the submission process. We grant 100% waivers to articles whose corresponding authors are based in Group A countries and 50% waivers to those who are based in Group B countries as classified by Research4Life (see: https://www.research4life.org/access/eligibility/ ). This section has its own submission website at https://mc.manuscriptcentral.com/e-videos .

## References

[1] Lee JH Kang DH Kim JY Endoscopic bilateral metal stent placement for advanced hilar cholangiocarcinoma: a pilot study of a newly designed Y stent Gastrointest Endosc 2007 66 364 369 10.1016/j.gie.2006.12.061 17643714

[2] Hwang JC Kim JH Lim SG Y-shaped endoscopic bilateral metal stent placement for malignant hilar obstruction: prospective long-term study Scand J Gastroenterol 2011 46 326 332 10.3109/00365521.2010.536253 21082874

[3] Lee TH Moon JH Kim JH Primary and revision efficacy of cross-wired metallic stents for endoscopic bilateral stent-in-stent placement in malignant hilar biliary strictures Endoscopy 2013 45 106 113 10.1055/s-0032-1325928 23212727

